# Supplementary material for: Modeling workplace contact networks: The effects of organizational structure, architecture, and reporting errors on epidemic predictions
Source: Netw Sci (Camb Univ Press). Author manuscript; Available in PMC 2015 Nov 30. (PMC4663701; doi:10.1017/nws.2015.22)
Supplement: Supple 1 [file NIHMS677490-supplement-Supple_1.pdf]

Supplementary Material to Modelling workplace contact networks: the effects of organizational structure, architecture, and reporting errors on epidemic predictions, published in *Network Science*

Gail E. Potter, Timo Smieszek, and Kerstin Sailer

April 24, 2015

A Comparison of reporting probability estimates to those in previous work

We compare the reporting probability estimates from our proportional odds model with angular distance to those from Smieszek *et al.* (2012) in table 1. The estimates obtained by the two different methods are extremely similar. The wide confidence interval for contacts lasting more than an hour is due to the fact that all contacts of this duration were reported with 100% consistency, so there is no variability with which to estimate the standard error of the reporting probability.

Table 1: Comparison of our reporting probability estimates to those in Smieszek et al. (Monday only)

Estimate	Angular Model	Smieszek et al.
0–5	0.56 [0.41, 0.69]	0.53
6–15	0.96 [0.84, 0.99]	0.96
16–60	0.93 [0.83, 0.99]	0.93
61–480	1.00 [0.00, 1.00]	1.00

B Results from proportional odds models with four different distance metrics

Table 2 shows results from proportional odds models with four different distance metrics. The model with angular distance metrics fits best according to the AIC.

Table 2: Coefficients for proportional odds models for contact duration, using four different distance metrics

	Metric		Topo		Angular		Axtopo	
Group 1	-0.32 (0.19)	.	-0.33 (0.19)	.	-0.18 (0.20)		-0.20 (0.20)	
Group 2	-0.07 (0.18)		-0.06 (0.18)		0.11 (0.19)		0.13 (0.20)	
Group mixing	3.41 (0.48)	***	3.49 (0.47)	***	3.42 (0.45)	***	3.39 (0.45)	***
Distance	-0.01 (0.02)		-0.01 (0.04)		-0.22 (0.08)	**	-0.20 (0.08)	*
Female	0.36 (0.21)	.	0.37 (0.21)	.	0.31 (0.21)		0.31 (0.21)	
Role mixing	0.79 (0.30)	**	0.83 (0.30)	**	0.60 (0.29)	*	0.63 (0.29)	*
Gender mixing	-0.21 (0.26)		-0.22 (0.26)		-0.18 (0.26)		-0.18 (0.26)	
Floor	1.12 (0.52)	*	1.23 (0.61)	*	-0.09 (0.68)		-0.33 (0.77)	
Shared projects	1.17 (0.28)	***	1.20 (0.28)	***	1.06 (0.28)	***	1.08 (0.28)	***
AIC	779.1		779.4		772.5		773.1	

Significance levels: *** $= p < 0.001$; ** $= p < 0.01$; * $= p < 0.05$; "." $= p < 0.10$

C Results from testing proportional odds model assumption

Table 3 compares log odds ratio estimates from logistic regression models fitted to contact duration, dichotomized at different cutoffs (0, 5, 15, or 60 minutes). Some estimates are effectively infinite, with infinite standard errors because either 0% or 100% cell counts were observed. The table suggests that while the proportional odds assumption probably does not hold perfectly, it is not unreasonable. Group mixing and distance coefficient estimates are remarkably similar, the two main effects of primary interest. Other coefficients vary somewhat, but differences are not statistically significant.

Table 3: Log odds ratio estimates and 95% confidence intervals at different dichotomizations of contact duration to test proportional odds model assumption, metric distance measure.

	Duration cutoff			
Effect	> 0	> 5	> 15	> 60
Group 1	-0.04	-0.02	-0.49	-0.65
	[-0.54, 0.46]	[-0.65, 0.60]	[-1.17, 0.20]	[-1.23, -0.06]
Group 2	-0.27	-0.05	-0.41	-0.58
	[-0.75, 0.22]	[-0.63, 0.53]	[-1.06, 0.24]	[-1.09, -0.06]
Group mixing	3.96	4.13	3.59	17.73
	[2.92, 5.01]	[2.57, 5.69]	[2.00, 5.18]	[NA, NA]
Distance	-0.01	-0.03	-0.02	-0.01
	[-0.04, 0.03]	[-0.07, 0.01]	[-0.06, 0.02]	[-0.06, 0.04]
Sex	-0.08	-0.04	0.33	0.19
	[-0.51, 0.35]	[-0.53, 0.44]	[-0.21, 0.87]	[-0.47, 0.86]
Role	0.93	1.52	1.76	-0.17
	[0.35, 1.51]	[0.86, 2.18]	[1.01, 2.51]	[-1.28, 0.94]
Gender mixing	-0.12	-0.29	-0.41	0.7
	[-0.66, 0.41]	[-0.91, 0.33]	[-1.12, 0.30]	[-0.24, 1.64]
Same floor	1.57	16.82	16.62	16.93
	[0.40, 2.74]	[NA, NA]	[NA, NA]	[NA, NA]
Shared projects	3.78	2.15	2.39	1.42
	[1.40, 6.15]	[0.90, 3.39]	[1.23, 3.55]	[0.32, 2.53]

D Additional fits of proportional odds models

Table 4: Coefficients (SEs) for proportional odds models for five days of the week, using angular distance metric

Intercepts	Monday		Tuesday		Wednesday		Thursday		Friday	
0	2.65 (1.04)	*	3.37 (0.88)	**	3.11 (1.14)	*	4.25 (0.98)	**	2.98 (1.4)	*
1-5	3.87 (1.04)	***	4.23 (0.89)	***	4.41 (1.13)	***	5.01 (0.98)	***	3.33 (1.40)	***
6-15	4.58 (1.04)	***	4.82 (0.89)	***	5.01 (1.13)	***	5.51 (0.99)	***	3.76 (1.40)	***
16-60	6.22 (1.08)	***	6.44 (0.93)	***	6.78 (1.15)	***	6.90 (1.00)	***	5.55 (1.41)	***
Group 1	-0.18 (0.20)		0.40 (0.18)	*	0.28 (0.18)		0.07 (0.18)		0.29 (0.31)	
Group 2	0.11 (0.19)		0.17 (0.20)		- 0.09 (0.18)		0.08 (0.19)		0.06 (0.33)	
Group mixing	3.42 (0.45)	***	2.75 (0.39)	***	4.54 (0.64)	***	3.94 (0.49)	***	3.00 (0.48)	***
Distance	-0.22 (0.08)	*	-0.13 (0.07)	.	-0.29 (0.09)	**	-0.16 (0.07)	*	-0.15 (0.11)	
Female	0.31 (0.21)		-0.09 (0.17)		0.29 (0.20)		0.02 (0.19)		- 0.02 (0.27)	
Role mixing	0.60 (0.29)	.	0.29 (0.25)		0.79 (0.30)	*	0.98 (0.24)	**	1.35 (0.37)	**
Gender mixing	-0.18 (0.26)		0.26 (0.22)		0.27 (0.25)		0.01 (0.22)		-0.50 (0.31)	
Floor	-0.09 (0.68)		-0.14 (0.56)		-0.76 (0.69)		0.63 (0.62)		-0.61 (0.79)	
Shared projects	1.06 (0.28)	**	1.62 (0.23)	***	1.28 (0.23)	***	0.82 (0.22)	**	0.65 (0.26)	*

E Multinomial logit model

E.0.1 Multinomial logit model likelihood

In this model we predict both contact and contact duration as a function of covariates. We use a multinomial logit model to estimate the probability of each of the four duration categories, or a fifth category, non-contact. We will now re-define our notation to reflect the inclusion of non-contact as a duration category. Define $\pi_k(x) = P(D_{ij} = d_k | X_{ij} = x)$, for $k = 0, \ldots, 4$ (representing categories 0, 1-5, 6-15, 16-60, and 61+ minutes). Let X_{ij} denote individual-level and dyadic covariates in our model. Again we let $\boldsymbol{D}$ denote the matrix of contact durations (after removing inconsistencies in duration reports) with non-contacts having duration zero. Using non-contact as the baseline duration category, the multinomial model is defined by Agresti (2002):

$$\log \frac{P(D_{ij} = d_k | X_{ij} = \mathbf{x})}{P(D_{ij} = d_1 | X_{ij} = \mathbf{x})} = \alpha_k + \beta_k^T \mathbf{x}, \text{ for } k = 1, 2, 3, 4$$

From this we obtain:

$$P(D_{ij} = d_k | X_{ij} = \mathbf{x}) = \frac{e^{\alpha_k + \beta_k^T \mathbf{x}}}{1 + \sum_{h=1}^{4} e^{\alpha_h + \beta_h^T \mathbf{x}}}$$

Because the probabilities must sum to one, $P(D_{ij} = d_0 | X_{ij} = x) = 1 - \sum_{h=1}^{4} e^{\alpha_h + \beta_h^T \mathbf{x}}$.

By applying our assumptions, rules of conditional probability, and the Law of Total Probability, we find that the joint likelihood of $\boldsymbol{D}$ and $\boldsymbol{C}$ is:

$$P(C_{ij} = 1, C_{ji} = 1, D_{ij} = d_k) = P(D_{ij} = d_k) p_k^2$$

$$P(C_{ij} = 1, C_{ji} = 0, D_{ij} = d_k) = P(D_{ij} = d_k) p_k (1 - p_k)$$

$$P(C_{ij} = 0, C_{ji} = 0, D_{ij} = 0) = P(D_{ij} = 0) + \sum_{k=1}^{4} P(D_{ij} = d_k)(1 - p_k)^2$$

Then the probability of the observed data is:

$$P(\boldsymbol{C} = \boldsymbol{c}, \boldsymbol{D} = \boldsymbol{d}) = \prod_{i=1}^{n} \prod_{j=i+1}^{n} P(C_{ij} = c_{ij}, C_{ji} = c_{ji}, D_{ij} = d_k)$$

We maximize the log likelihood to estimate $\boldsymbol{\alpha}$, $\boldsymbol{\beta}$, and $\mathbf{p}$ using the `trust` function in R and computed standard errors by inverting the Fisher information matrix (Geyer, 2009).

F Goodness of fit to assess modelling of transitivity

Figure 1 compares goodness of fit diagnostics for two models in order to assess how well our model captured transitivity present in the network. The first model is our ERGM with angular distance, fit to a nondirectional binary network created by assuming that contact between two individuals occurred if it was reported by at least one of the two. The second model is the same ERGM, but also including a geometrically weighted edgewise shared partners (gwesp) term with alpha = 0.5. The box plots show network statistics for networks simulated from each model, while the solid line shows network statistics for the actual data. The figures show that our model does a good job representing the degree distribution and the minimum geodesic distance of the network, but overestimates the proportion of edges with 2–3 shared partners, and underestimates the proportion of edges with 6–8 shared partners. The model with the added gwesp term mostly corrects this problem.

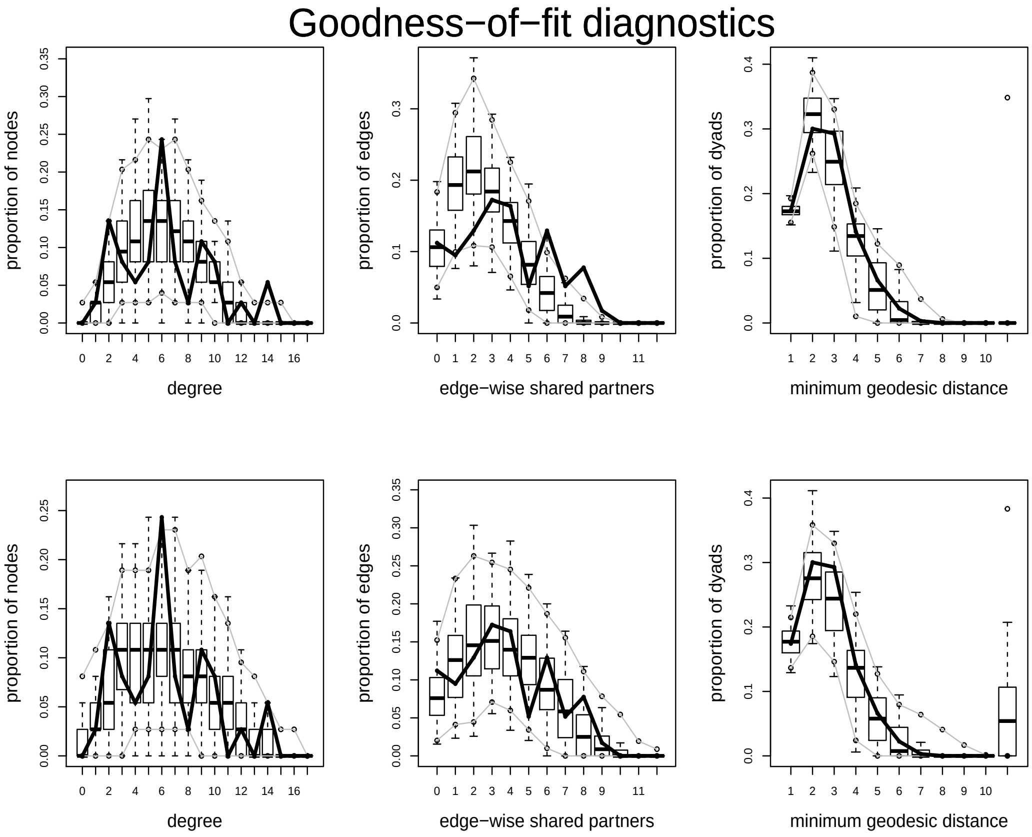

Figure 1: Goodness of fit diagnostics for our model (top) without adjusting for reporting errors, compared to those for an extension of model which also includes a gwesp(0.5) term to capture transitivity.

F.1 Multinomial logit model likelihood results

Table 5 shows coefficient estimates from the multinomial logit model with four distance metrics. Coefficients are interpreted as follows: The odds of a 1–5 minute contact relative to no contact increases by a factor of $e^{3.24} = 26$ if two people are in the same research group, controlling for other variables in the model. The odds of a 16–60 minute contact relative to no contact decreases by a factor of $e^{-0.05} = 0.95$ for each unit increase in metric distance between their workstations, controlling for other variables in the model. Some coefficients do not have finite standard errors because of zero or 100% cell counts. For example, all reported 16–60 and 60+ minute contacts were on the same floor. The floor coefficient for these categories should be infinite, but is estimated as a very large number (after exponentiation). All reported 61+ minute contacts were among members of the same research group, resulting in an infinite coefficient for group mixing. The set of predictor variables in the multinomial model that we fit differs from our full model in the text in that the shared projects is excluded. However, inclusion of this variable would only amplify the estimation problems caused by a large number of parameters being estimated with several cases of small cell counts.

We include in this section estimates from the proportional odds model so the reader may compare them to the multinomial model.

Table 5: Multinomial model estimates (SEs)

	Metric		Angular		Topo		Axtopo	
1–5 minutes								
Int.	-4.48 (0.94)	***	-2.28 (1.05)	*	-4.85 (1.02)	***	-2.36 (1.18)	*
Group 1	-0.01 (0.2)		0.15 (0.21)		-0.02 (0.2)		0.11 (0.21)	
Group 2	0.29 (0.2)		0.5 (0.22)	*	0.28 (0.2)		0.49 (0.23)	*
Group Mixing	3.24 (0.44)	***	3.22 (0.41)	***	3.28 (0.43)	***	3.19 (0.41)	***
Distance	0 (0.02)		-0.21 (0.1)	*	0.02 (0.04)		-0.16 (0.09)	.
Female	-0.13 (0.21)		-0.15 (0.21)		-0.13 (0.21)		-0.15 (0.21)	
Role Mixing	0.42 (0.31)		0.24 (0.32)		0.45 (0.31)		0.29 (0.31)	
Gender Mixing	-0.3 (0.26)		-0.28 (0.26)		-0.3 (0.26)		-0.29 (0.26)	
Floor	0.22 (0.48)		-1.15 (0.72)		0.42 (0.57)		-1.2 (0.84)	
6–15 minutes								
Int.	-6.6 (1.47)	***	-2.34 (1.42)		-6.6 (1.58)	***	-1.91 (1.53)	
Group 1	-0.08 (0.26)		0.31 (0.28)		-0.08 (0.26)		0.25 (0.27)	
Group 2	0.37 (0.25)		0.86 (0.28)	**	0.4 (0.26)		0.89 (0.28)	**
Group Mixing	3.63 (0.79)	***	3.78 (0.78)	***	3.68 (0.79)	***	3.7 (0.78)	***
Distance	-0.02 (0.02)		-0.55 (0.11)	***	-0.05 (0.05)		-0.48 (0.1)	***
Female	0.21 (0.26)		0.09 (0.27)		0.21 (0.26)		0.09 (0.26)	
Role Mixing	0.9 (0.37)	*	0.48 (0.37)		0.91 (0.37)	*	0.53 (0.37)	
Gender Mixing	-0.23 (0.33)		-0.17 (0.34)		-0.24 (0.33)		-0.17 (0.34)	
Floor	1.21 (0.88)		-1.84 (1.06)	.	1.05 (0.98)		-2.4 (1.17)	*
16–60 minutes								
Int.	-19.76 (NA)		-18.94 (NA)		-19.94 (NA)		-18.23 (NA)	
Group 1	-0.12 (0.23)		0.09 (0.24)		-0.13 (0.23)		0.05 (0.23)	
Group 2	-0.09 (0.23)		0.26 (0.24)		-0.04 (0.23)		0.28 (0.24)	
Group Mixing	3.72 (0.78)	***	4.05 (0.75)	***	3.75 (0.78)	***	4 (0.75)	***
Distance	-0.05 (0.02)	**	-0.39 (0.1)	***	-0.1 (0.04)	*	-0.35 (0.1)	***
Female	1.1 (0.4)	**	1.05 (0.41)	*	1.12 (0.4)	**	1.05 (0.41)	*
Role Mixing	1.58 (0.38)	***	1.53 (0.37)	***	1.6 (0.38)	***	1.56 (0.37)	***
Gender Mixing	-1.31 (0.45)	**	-1.38 (0.45)	**	-1.33 (0.45)	**	-1.37 (0.45)	**
Floor	14.49 (NA)		13.8 (NA)		14.5 (NA)		12.99 (NA)	
61+ minutes								
Int.	-26.51 (NA)		-52.12 (6.45)	***	-29.14 (NA)		-24.45 (NA)	
Group 1	-0.74 (0.3)	*	-0.5 (0.31)		-0.72 (0.3)	*	-0.53 (0.31)	.
Group 2	-0.61 (0.26)	*	-0.27 (0.28)		-0.56 (0.26)	*	-0.21 (0.29)	
Group Mixing	13.72 (126.44)		42.44 (10.25)	***	14.7 (116.34)		14.09 (NA)	
Distance	-0.04 (0.03)		-0.35 (0.15)	*	-0.03 (0.06)		-0.34 (0.14)	*
Female	0.38 (0.34)		0.3 (0.34)		0.35 (0.34)		0.31 (0.34)	
Role Mixing	0.31 (0.54)		0.28 (0.51)		0.53 (0.54)		0.27 (0.52)	
Gender Mixing	0.53 (0.48)		0.47 (0.48)		0.47 (0.48)		0.48 (0.48)	
Floor	11.83 (NA)		9.15 (NA)		12.86 (NA)		9.78 (NA)	
AIC	1478		1453		1480		1456	

Significance levels: *** $= p < 0.001$; ** $= p < 0.01$; * $= p < 0.05$; "." $= p < 0.10$

Table 6: Coefficients (SEs) for proportional odds models for contact duration, using four different distance metrics

	Metric		Angular		Topo		Axtopo	
Group 1	-0.32 (0.19)	.	-0.18 (0.20)		-0.33 (0.19)	.	-0.20 (0.20)	
Group 2	-0.07 (0.18)		0.11 (0.19)		-0.06 (0.18)		0.13 (0.20)	
Group mixing	3.41 (0.48)	***	3.42 (0.45)	***	3.49 (0.47)	***	3.39 (0.45)	***
Distance	-0.01 (0.02)		-0.22 (0.08)	**	-0.01 (0.04)		-0.20 (0.08)	*
Female	0.36 (0.21)	.	0.31 (0.21)		0.37 (0.21)	.	0.31 (0.21)	
Role mixing	0.79 (0.30)	**	0.60 (0.29)	*	0.83 (0.30)	**	0.63 (0.29)	*
Gender mixing	-0.21 (0.26)		-0.18 (0.26)		-0.22 (0.26)		-0.18 (0.26)	
Floor	1.12 (0.52)	*	-0.09 (0.68)		1.23 (0.61)	*	-0.33 (0.77)	
Shared projects	1.17 (0.28)	***	1.06 (0.28)	***	1.20 (0.28)	***	1.08 (0.28)	***
AIC	779.1		772.5		779.4		773.1	

Significance levels: *** = $p < 0.001$; ** = $p < 0.01$; * = $p < 0.05$; "." = $p < 0.10$

Table 7: Coefficients [95% Confidence Intervals] for multinomial model with no floor effect and two largest duration categories collapsed

METRIC MODEL	1-5 mins		6-15 mins		16+ mins	
Effect	Est.	95% CI	Est.	95% CI	Est.	95% CI
Intercept	-4.16	[-5.66, -2.66]	-5.22	[-7.47, -2.97]	-4.07	[-6.06, -2.07]
Group 1	-0.03	[-0.42, 0.37]	-0.13	[-0.66, 0.41]	-0.44	[-0.84, -0.05]
Group 2	0.31	[-0.06, 0.69]	0.43	[-0.09, 0.95]	-0.30	[-0.68, 0.08]
Group Membership	3.23	[2.36, 4.11]	3.68	[2.09, 5.28]	4.14	[2.61, 5.67]
Distance	0	[-0.03, 0.02]	-0.04	[-0.08, -0.01]	-0.07	[-0.10, -0.04]
Sex	-0.12	[-0.53, 0.29]	0.24	[-0.27, 0.75]	0.68	[0.18, 1.19]
Role mixing	0.43	[-0.19, 1.06]	0.86	[0.13, 1.59]	1.13	[0.49, 1.76]
Sex mixing	-0.29	[-0.81, 0.23]	-0.16	[-0.82, 0.49]	-0.49	[-1.10, 0.12]
TOPO MODEL	1-5 mins		6-15 mins		16+ mins	
Effect	Est.	95% CI	Est.	95% CI	Est.	95% CI
Int.	-4.27	[-5.7, -2.84]	-5.27	[-7.47, -3.07]	-4.26	[-6.24, -2.28]
Group 1	-0.03	[-0.43, 0.37]	-0.10	[-0.63, 0.43]	-0.42	[-0.81, -0.03]
Group 2	0.32	[-0.06, 0.70]	0.44	[-0.08, 0.96]	-0.26	[-0.64, 0.12]
Group Mixing	3.28	[2.43, 4.13]	3.68	[2.10, 5.26]	4.18	[2.65, 5.71]
Distance	0	[-0.05, 0.05]	-0.09	[-0.16, -0.02]	-0.14	[-0.20, -0.08]
Female	-0.12	[-0.53, 0.29]	0.24	[-0.27, 0.75]	0.69	[0.19, 1.19]
Role Mixing	0.43	[-0.19, 1.05]	0.85	[0.12, 1.58]	1.13	[0.49, 1.77]
Gender Mixing	-0.29	[-0.81, 0.23]	-0.19	[-0.85, 0.47]	-0.53	[-1.14, 0.08]
ANGULAR MODEL	1-5 mins		6-15 mins		16+ mins	
Effect	Est.	95% CI	Est.	95% CI	Est.	95% CI
Int.	-3.63	[-4.90, -2.36]	-4.11	[-6.13, -2.09]	-4.15	[-6.01, -2.29]
Group 1	0.09	[-0.33, 0.51]	0.26	[-0.28, 0.8]	-0.13	[-0.53, 0.27]
Group 2	0.31	[-0.06, 0.68]	0.70	[0.18, 1.22]	0.01	[-0.36, 0.38]
Group Mixing	3.06	[2.28, 3.84]	3.53	[2.03, 5.03]	4.37	[2.89, 5.85]
Distance	-0.08	[-0.18, 0.02]	-0.45	[-0.63, -0.27]	-0.40	[-0.54, -0.26]
Female	-0.16	[-0.57, 0.25]	0.09	[-0.44, 0.62]	0.57	[0.07, 1.07]
Role Mixing	0.30	[-0.31, 0.91]	0.53	[-0.20, 1.26]	1.09	[0.47, 1.71]
Gender Mixing	-0.28	[-0.80, 0.24]	-0.18	[-0.85, 0.49]	-0.59	[-1.20, 0.02]
AXTOPO MODEL	1-5 mins		6-15 mins		16+ mins	
Effect	Est.	95% CI	Est.	95% CI	Est.	95% CI
Int.	-3.79	[-5.05, -2.53]	-4.32	[-6.32, -2.32]	-4.19	[-6.04, -2.34]
Group 1	0.06	[-0.35, 0.47]	0.18	[-0.35, 0.71]	-0.17	[-0.57, 0.23]
Group 2	0.31	[-0.06, 0.68]	0.69	[0.18, 1.20]	0.03	[-0.35, 0.41]
Group Mixing	3.09	[2.30, 3.88]	3.46	[1.96, 4.96]	4.24	[2.76, 5.72]
Distance	-0.05	[-0.13, 0.03]	-0.36	[-0.52, -0.2]	-0.35	[-0.48, -0.22]
Female	-0.15	[-0.56, 0.26]	0.10	[-0.42, 0.62]	0.57	[0.07, 1.07]
Role Mixing	0.34	[-0.27, 0.95]	0.60	[-0.13, 1.33]	1.11	[0.49, 1.73]
Gender Mixing	-0.29	[-0.81, 0.23]	-0.18	[-0.85, 0.49]	-0.59	[-1.2, 0.02]

References

Agresti, A. (2002). *Categorical Data Analysis*. 2nd edn. Wiley Series in Probability and Statistics. Wiley-Interscience.

Geyer, Charles J. (2009). *trust: Trust region optimization*. R package version 0.1-2.

Smieszek, Timo, Burri, Elena U., Scherzinger, Robert, & Scholz, Roland W. (2012). Collecting close-contact social mixing data with contact diaries: reporting errors and biases. *Epidemiology and Infection*, **140**(4), 744–752.
